# Society Meetings

**Published:** 1917-03

**Authors:** 


### SOCIETY MEETINGS

---

Brief reports and announcements of meetings of societies of Western New York. and those of general scope, are requested from Secretaries. Copy should be on hand the fifteenth of the month. Full transactions will be published at cost of composition.

---

**DOCTOR: Is your Society properly represented here? If not, please bring our standing announcement to the attention of the members.**

---

**Excerpt, from 1916 Annual Report of Board of Censors of the Medical Society of the County of Erie,** by J. D. Bonnar, chairman. That much has been written and said about the "division of fees" question, is well known to us all. Its cognate associate, in the ethical field, is the fee charged for surgical work. Not that we should lessen, in the least degree, the merits of any one of the vast variety of operations, justly performed for human relief. That is not our object, but rather that there be retained a just parity between the surgical and medical service demanded for such relief of the sick. Forsooth, if the surgical aid absorbs all the means at the disposal of the patient, or nearly all, the physician has short shrift. Now it is apparent, upon close study, that there is right in that field a vital lack of strict equity. To rectify this error, I personally maintain that the fee, which is paid for the surgical work should be submitted to the opinion of the attending physician. Thus his sense of the eternal fitness of things would give an excellent impression, while standardising the operation with the patient's ability to pay. The physician meanwhile having an appreciable equity in the case, must also have his equity protected. This analysis will not only give the patient his choice of physicians but give him also the intelligent council of his physician, in deliberations upon any operation, that they may have in prospect. It will likewise be conceded that the diagnosis is a vital first principle, in any case that suggests surgical relief. This responsibility rests chiefly upon the attending physician. Thus is his claim for a parity, in the care of the case, both prior and subsequent to any surgical aid and hence likewise is his right to fair and equitable compensation, justified. In all this reasoning, upon a vital and rational question, we are not doubting the justice of fair compensation for surgical work, but simply demonstrating the parity of responsibility and knowledge demanded of the physician, with that of the surgeon. Hence, in the matter of compensation the money, available for the treatment of the case in hand, must be conserved so that each man in the care of the case, surgically, or medically, is paid out to meet the equitable rights of each—not merely to pay the surgeon. I am not speaking for personal interests, as I have always done my own surgical work—"A capite ad calcem"—from trephining the skull, to excision of a toe. I therefore am entitled, as a physician and surgeon, to speak in this matter, for full equity and even-handed justice.

---

At the regular meeting of the **Medical Society of the County of Erie**, held at the Buffalo Medical College, Feb. 19, 1917, Dr. Irving W. Potter, presiding, the following were

Reinstated: Dr. James P. Barr, Dr. B. Jacobson, Dr. T. V. Bauer, Dr. W. W. Ross.

Elected: Dr. S. Barone, Dr. B. Bukowski, Dr. F. S. Miller, Dr. L. N. La Mantia, Dr. P. H. J. Buckley, Dr. C. C. Hoffman.

This meeting was devoted entirely to the consideration of the Mills bill now before the legislature, relative to Compulsory Health Insurance. It was discussed from every angle by many of the members present and as a result the following resolution was unanimously adopted:

Resolved, That the Medical Society of the County of Erie, after due and careful consideration, is diametrically opposed to the passage of the proposed Compulsory Health Insurance act on the ground that the said proposed measure is decidedly inimical to the best interests of the public at large and the medical profession.

A postal card referendum had likewise been conducted, the cards being sent to the members with the notice of the meeting. Of the number sent out 435 were returned. Out of this number 12 expressed themselves as in favor of the bill and 423 as opposed to it.

The Society was also notified that the Buffalo Medical & Surgical League, Gross Medical Club of Buffalo, and the Twin City Academy of Medicine of Tonawanda, N. Y., were also opposed to this measure.

The Secretary also presented resolutions against the Mills bill adopted by the Medical Society of the County of Schenectady, the Flat Bush Medical Society of Brooklyn, N. Y., and by the New York Chamber of Commerce.

The following resolution was also adopted:

Resolved, That the Medical Society of the County of Erie most emphatically disapproves and condemns the extra legal if not illegal endorsement by the Council of the Medical Society of the State of New York of a measure not fully approved by the body of the profession, either in principal or in policy, and while it is still under consideration and discussion by those most vitally affected by its provisions.

FRANKLIN C. GRAM, M. D., Secretary.

---

**The Gross Medical Club** held its annual meeting on Friday, Feb. 18th, being entertained by Dr. Geo. F. Cott. The election of officers for the coming year resulted: Dr. Jas. E. King president, Dr. Byron E. Smith of Angola vice-president, Chester C. Cott of Buffalo secretary.

Dr. W. A. Phelps of East Aurora read a very interesting paper on Trichinosis, reporting a series of 16 cases occuring at Elma, N. Y. The earliest appearance of the symptoms was seven days in one case, the longest incubation being 28. All the cases were traceable to one hog that was owned by a farmer at Elma. The sausage, which was the food consumed, had been eaten raw by the different patients, all of which had visited the house of the farmer owning the hog, or had some of the sausage sent them.

On motion of Dr. J. Henry Dowd a resolution was unanimously passed condemning the proposed health insurance bill (Mills), and Dr. Grover W. Wende was appointed as a delegate to go to Albany for the hearing and represent the Gross Club in opposition.

---

**The Federated Alumni of the University of Buffalo** held the third annual dinner Feb. 22 at the Hotel Statler, Buffalo.

---

**The Medical Society of the County of Chemung** held its regular annual meeting Dec. 19. President's address, R. H. V. Dann, M. D.; Internal use of water, J. C. Fisher, M. D. Officers elected: President, Dr. Clyde Carey; Vice-President,

Dr. Alexander Mark; Secretary, Dr. Mabel Flood; Treasurer, Dr. H. W. Fudge; board of censors, Drs. R. G. Loop, William Brady, C. G. R. Jennings. Amendment to section 1, chapter 10 in by-laws concerning increase in dues was voted upon.

---

**The Dental Alumni of the University of Buffalo** held its 17th annual meeting Feb. 16 and 17. As customary, much of the time was devoted to clinics. Those in crown and bridge work were conducted by Dr. Edward B. Spaulding of Detroit, Dr. Marcus L. Ward, Dean of the College of Dentistry of the University of Michigan, Ann Arbor, and Dr. E. T. Tinker of Minneapolis. Officers were elected as follows: President, E. J. Farmer, Buffalo; Vice-president, C. A. Pankow, Buffalo; Rec. Sec., J. L. Clement, Buffalo; Treasurer, W. R. Montgomery.

---

At the meeting of the **Staff of the Buffalo Hospital of the Sisters of Charity,** Feb. 16, the following officers were elected: President, Laurence George Hanley, M. D., LL.D.,; Vice-president, Emil Tobie, M. D.; Sec., Frank Abbott, M. D.

**The Southern Gastro-Enterological Assn.** was organized in Atlanta this winter, representing 17 southern states. Annual meetings will be held. Officers elected were as follows: Pres. J. C. Johnson, Atlanta; V. P., J. T. Rogers, Savannah; Sec.-Treas., Marvin H. Smith, Jacksonville; Council, S. K. Simon, New Orleans, G. M. Niles, Atlanta, Seale Harris, Birmingham; Committee on Admission and Ethics, George C. Mizell, Atlanta, J. E. Knighton, Shreveport, J. B. Fitts, Atlanta.

---

**The Elmira Academy of Medicine** held its regular meeting Feb. 7. Program: Some Gynecological Cases, Dr. C. F. Abbott; Focal Infection, Dr. J. Lee Kinner. R. B. Howland, M. D., President; O. J. Bowman, M. D., Secretary.

---

**The Rochester Academy of Medicine Section II,** held its regular meeting Feb. 14. Order: Genito-Urinary Infections, Dr. A. H. Paine. Five minute discussions: In Orthopedics, Dr. H. L. Prince; In Obstetrics, Dr. J. P. Quigley; in Pediatrics, Dr. N. G. Orchard; in Neurology, Dr. E. L. Hanes; in Cardio-Vascular Diseases, Dr. C. C. Sutter; in Rectal Diseases Dr. C. Reitz. General discussion. William I. Dean, M. D., Chairman; Leonard W. Jones, M. D., Secretary.

---

**The Buffalo Academy of Medicine** has held the following meetings since last reported: Section of Surgery, Feb. 7, discussion of certain borderline problems: A, Cholecystectomy vs. Cholecystostomy; B, Treatment of Gastric and Duodenal Ulcers; C, Relationship of the Thyroid to Exophthalmic Goitre. Stereopticon and motion pictures illustrating certain points in Surgcial Technic, Dr. George W. Crile, Cleveland, Ohio. Dr. F. M. O'Gorman, Chairman; Dr. J. B. Cross, Secretary.

Section of Medicine, Feb. 14. Pneumococcic Peritonitis in Infancy and Childhood, Isaac Abt, M. D., Prof. of Diseases of Children, Northwestern University Medical School, Chicago. E. A. Sharp, M. D., Chairman; H. Mead, M. D., Secretary.

Obstetric Section, Feb. 21. Labor in Subnormal Pelves, Dr. F. C. Goldsborough. Discussion will be opened by Dr. Van Peyma. I. W. Potter, M. D., Chairman; H. K. DeGroat, M. D., Secretary.

